# Core-Tunable Dendritic Polymer: A Folate-Guided Theranostic Nanoplatform for Drug Delivery Applications

**DOI:** 10.1021/acsomega.4c02258

**Published:** 2024-07-06

**Authors:** Neelima Koti, Trishna Timalsena, Kajal Kajal, Caleb Worsley, Adam Worsley, Paul Worsley, Carissa Sutton, Tuhina Banerjee, Santimukul Santra

**Affiliations:** † Department of Chemistry and Biochemistry, Missouri State University, 901 S. National Avenue, Springfield, Missouri 65897, United States; ‡ Department of Chemistry, Pittsburg State University, 1701 S. Broadway Street, Pittsburg, Kansas 66762, United States

## Abstract

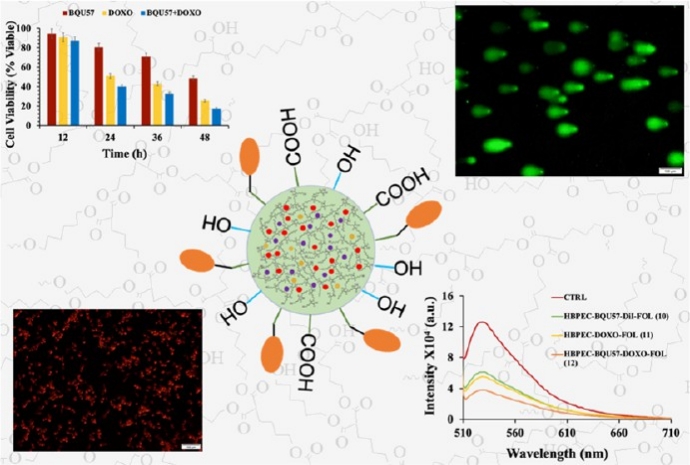

Clinical application of anticancer drugs is mostly limited due to their hydrophobic nature, which often results in lower bioavailability and lesser retention in systemic circulation. Despite extensive research on the development of targeted drug delivery systems for cancer treatment, delivery of hydrophobic therapeutic drugs to tumor cells remains a major challenge in the field. To address these concerns, we have precisely engineered a new hyperbranched polymer for the targeted delivery of hydrophobic drugs by using a malonic acid-based A_2_B monomer and 1,6-hexanediol. The choice of monomer systems in our design allows for the formation of higher molecular weight polymers with hydrophobic cavities for the efficient encapsulation of therapeutic drugs that exhibit poor water solubility. Using several experimental techniques such as NMR, differential scanning calorimetry (DSC), thermogravimetric analysis (TGA), Fourier transform-infrared (FT-IR), and gel permeation chromatography (GPC), the synthesized polymer was characterized, which indicated its dendritic structure, thermal stability, and amorphous nature, making it suitable as a drug delivery system. Following characterizations, theranostic nanoplatforms were formulated using a one-pot solvent diffusion method to coencapsulate hydrophobic drugs, BQU57 and doxorubicin. To achieve targeted delivery of loaded therapeutic drugs in A549 cancer cells, the surface of the polymeric nanoparticle was conjugated with folic acid. The therapeutic efficacy of the delivery system was determined by various cell-based *in vitro* experiments, including cytotoxicity, cell internalizations, reactive oxygen species (ROS), apoptosis, migration, and comet assays. Overall, findings from this study indicate that the synthesized dendritic polymer is a promising carrier for hydrophobic anticancer drugs with higher biocompatibility, stability, and therapeutic efficacy for applications in cancer therapy.

## 1 Introduction

Lung cancer is the second most diagnosed cancer in the United States for both men and women, with an estimated 238,340 new cases and 127,070 deaths predicted in 2023.^1,2^ Non-small cell lung cancer (NSCLC) comprises 85% of cases, compared with small cell lung cancer (SCLC). Surgery combined with immunotherapy, chemotherapy, and radiotherapy are common existing therapies for NSCLC.^3^ Small molecule-based chemotherapy involves the use of therapeutic drugs that target specific molecular pathways, including cisplatin, doxorubicin, paclitaxel, and others. However, the use of these treatments is limited due to severe toxicity, multidrug resistance, and poor survival outcomes associated with the nonspecific distributions.^4,5^ Nanomedicine-based therapy encompasses utilizing nanoscale materials to deliver therapeutic agents directly to the tumor site. This reduces the noncancerous cells’ exposure to highly toxic chemotherapeutic drugs, thereby reducing side effects such as hair loss, nausea, and mouth sores.^6^ Engineered nanomedicine with improved drug solubility and stability allows the controlled release of drugs, which increases the efficacy and reduces side effects.

Various nanomaterials such as metallic nanoparticles, including iron oxide,^7−10^ gold,^11−15^ cerium oxide,^16−19^ quantum dots,^20−24^ and nonmetallic nanoparticles, including micelles,^25−27^ liposomes,^28−30^ carbon nanotubes,^31−33^ graphene oxide,^34−37^ silica nanoparticles,^38,39^ and polymeric nanoparticles,^40−43^ have been reported as theranostic drug delivery systems. Metallic nanoparticles have shown tremendous potential for cancer imaging and treatment because of their several desirable properties, such as magnetic, thermal, light emission, optical, and antioxidant properties. Their smaller size allows for easy penetration, while a large surface area-to-volume ratio enables efficient loading and release of therapeutic agents, which enhances treatment efficacy and minimizes systemic toxicity.^44^ In addition, when functionalized with targeting ligands, such as antibodies or peptides, it selectively delivers drugs to cancer cells, reducing off-target effects. For instance, iron oxide nanoparticles with unique magnetic properties enable magnetic resonance imaging (MRI) for real-time monitoring of drug distribution and response. Cerium oxide nanoparticles (nanoceria) with unique redox properties enhance oxidative stress in tumors that play a significant role in cancer progression.^45−47^ Quantum dots, on the other hand, are semiconductor nanoparticles with tunable fluorescence properties based on their size and composition, making them suitable for imaging applications.

A broad range of polymers have been used in formulating nanomedicines, which can be classified as linear or branched based on their architecture and functionality. Linear polymers have emerged as versatile nanocarriers for delivering therapeutic drugs due to their ease of synthesis and tunable properties. For instance, poly(lactic acid) (PLA) and poly(glycolic acid) (PGA) are biocompatible polymers, easily synthesized through the ring-opening polymerization of respective monomers.^48−52^ These structures are commonly used to formulate polymeric nanoparticles that encapsulate various therapeutic agents, such as paclitaxel,^53^ docetaxel,^54^ and doxorubicin,^55^ and are used as drug delivery systems. However, these polymers have limited applications due to inadequate surface functionality, low payload capacity, challenges in tailoring release kinetics, and low solubility. To minimize these limitations, dendritic polymers, including dendrimers and hyperbranched polymers, are introduced as drug delivery systems.^56−58^ These three-dimensional, highly branched, multifunctional polymeric structures have gained significant attention as efficient nanocarriers for encapsulating and delivering therapeutic drugs and imaging agents. Divergent and Convergent growth are the two main approaches for synthesizing dendrimers with precise control over dendrimers’ size, shape, and functionality. For instance, poly(amidoamine) (PAMAM) and poly(propylene imine) (PPI) dendrimers are synthesized using divergent growth methods and applied for drug delivery and other biomedical applications.^59^ However, the laborious, multistep synthesis and high cost of production limit their theranostic applications.

Unlike dendrimers, hyperbranched polymers (HBPs) are another class of three-dimensional polymers that can overcome these limitations for low-cost, one-step synthesis while providing enhanced properties such as enhanced drug loading capacity, broader size distribution, reduced cytotoxicity, and versatile surface functionalization.^60,61^ The classical functional AB_*x*_ monomer is commonly used to synthesize HBPs, in which A and B represent acidic and basic functional groups. Nie et al. synthesized hyperbranched poly(amine-ester) and polylactide copolymer (HPAE-*co*-PLA), which was used to nanoformulate and deliver 2-benzoylpyridine 4-ethyl-3-thiosemicarbazone (Bp4eT) for the treatment of lung cancer. In another study by Kolhe et al., ibuprofen was conjugated to hydroxyl terminal groups of hyperbranched polyglycerols, and a high dose of it was delivered to the A549 lung cancer cells by the intracellular hydrolysis of the ester bonds in the presence of lysosomal enzymes. Our previous research developed a proprietary A_2_B monomer from diethyl malonate and successfully reported the hyperbranched polyester (HBPE).^62^ This parent HBPE polymer was used to formulate nanomedicines for the delivery of paclitaxel to tumor cells. Next, the polarity of HBPE polymers’ cavities was tuned by copolymerizing the A_2_B monomer with trigol, which facilitated the delivery of a more hydrophilic drug, doxorubicin hydrochloride, to prostate cancer cells.^63^ These results indicated the importance of our parent HBPE polymers’ unique design and potential to foster research and development.

**Scheme 1:**
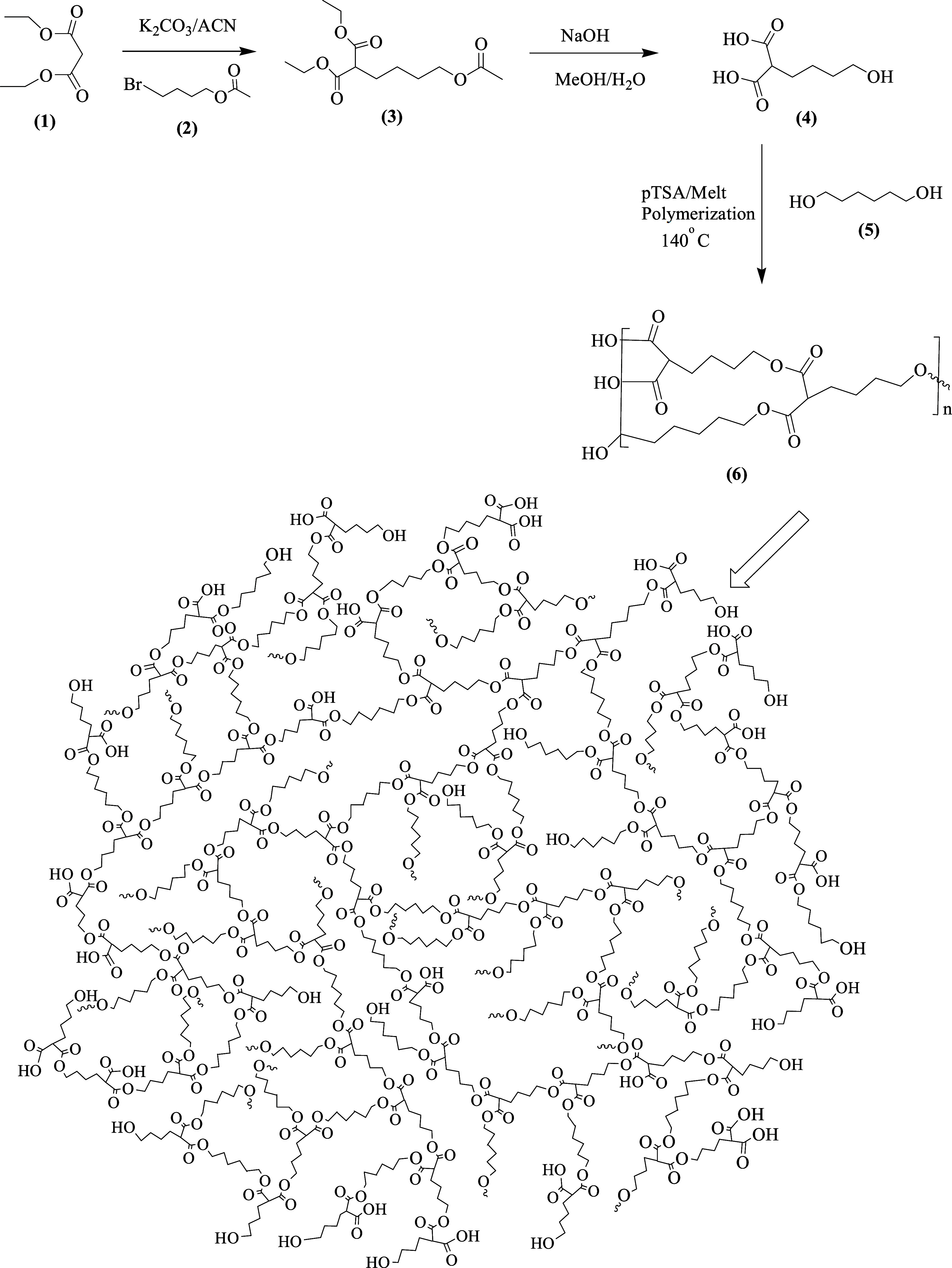
Synthesis of a Biocompatible Hyperbranched Polyester Copolymer (HBPEC) for Drug Delivery Applications

In the interest of further developing our HBPE platform, herein we demonstrate the synthesis of a new HBPE copolymer (HBPEC) with a hydrophobic polymeric cavity for encapsulating a variety of hydrophobic drugs. To do this, an A_2_B + B_2_ copolymerization technique is followed using our proprietary A_2_B monomer (**4**, Scheme 1) and 1,6-hexanediol. The inclusion of 1,6-hexanediol enhances hydrophobic–hydrophobic interactions between the cavity and the encapsulating hydrophobic drugs. The carboxylic acid groups in the A_2_B monomer provide functionality on the polymer surface (**6**, Scheme 1), allowing for easy targeting by ligand conjugation and ensuring the aqueous stability of nanoparticles in solution. Ester linkages in the polymer backbone and high molecular weight contribute to biocompatibility, degradation, and effective encapsulation of theranostic molecules. Combination therapy is an effective treatment where two or more therapeutic agents are used. It targets key pathways in a synergetic or additive manner while potentially reducing drug resistance and drug toxicity. In this work, we used hydrophobic doxorubicin, which inhibits the enzyme topoisomerase II followed by DNA damage,^64^ and a hydrophobic BQU57 drug, which is a Ral inhibitor.^65−67^ To demonstrate the ability of our HBPEC polymer to deliver hydrophobic drugs, both doxorubicin and BQU57 are coencapsulated and delivered for assessing antitumor activities on A549 cells. Together, these unique features would make our HBPEC polymer an ideal drug delivery platform to deliver a wide range of hydrophobic therapeutic drugs to tumor cells.

## 2 Results and Discussion

### 2.1 Polymer Synthesis and Characterizations

For synthesizing the HBPEC polymer, commercially available 1,6-hexanediol (**5**) and our previously reported^62^ A_2_B monomer (**4**) were used. Overall, the synthesis process began with the selective mono-C-alkylation of diethyl malonate (**1**) with 4-bromobutyl acetate (**2**) to obtain the corresponding A_2_B diester monomer (**3**). This was then hydrolyzed with sodium hydroxide to obtain the final A_2_B diacid monomer (**4**), followed by column purification and characterization using NMR and Fourier transform-infrared (FT-IR) spectroscopy. Next, to synthesize the HBPEC polymer (**6**, Scheme 1), the A_2_B monomer (**4**) and 1,6-hexanediol (**5**) were mixed in a 1:1 molar ratio with a catalytic amount (100:1) of freshly crystallized *p*TSA. The polymerization reaction was carried out at 140 °C for 2 h, and the accumulation of water vapor in the reaction vessel indicated the formation of ester bonds after the reaction between the acid and alcohol monomers. Afterward, the reaction was put under a medium vacuum (1.5 mmHg) to remove water vapor and continued polymerization for 8 h. The resulting copolymer (**6**) was purified using a mixed-solvent (DMSO/deionized (DI) water) precipitation method. The purified polymer was centrifuged, washed with DI water, and dried under high vacuum at 40 °C. The resulting copolymer (**6**) appeared as a highly viscous transparent material and was soluble in dimethyl sulfoxide, tetrahydrofuran, and chloroform.

**Figure 1:**
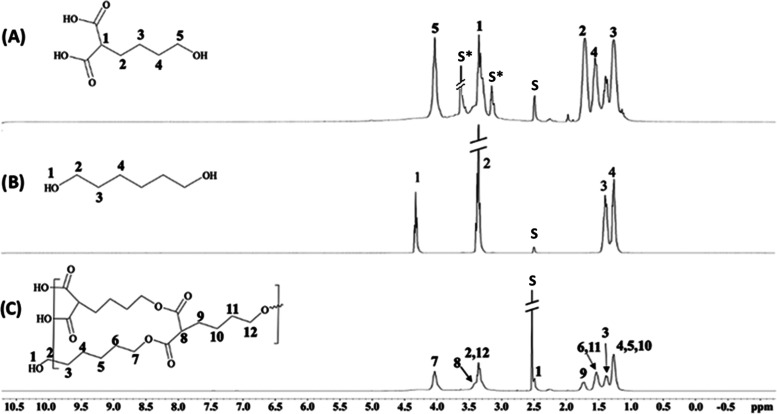
^1^H NMR spectra of the (A) A_2_B monomer, (B) 1,6-hexanediol, and (C) HBPEC. S = deuterated solvent DMSO-*d*_6_ and S* = solvent residue.

**Figure 2:**
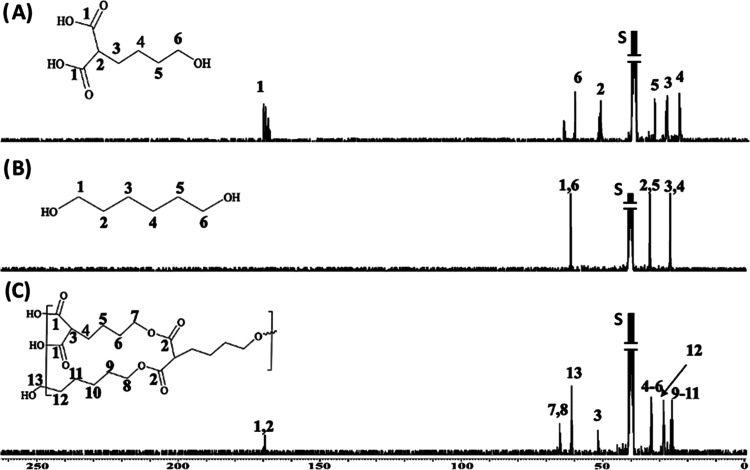
^13^C NMR spectra of the (A) A_2_B monomer, (B) 1,6-hexanediol, and (C) HBPEC. S = DMSO-*d*_6_ was used as the deuterated solvent.

For structural analysis, NMR and FT-IR spectroscopic methods were used. The ^1^H NMR of monomers **4** and **5** were labeled at their respective positions, as shown in Figure 1, using a 300 MHz NMR spectrometer. The HBPEC polymer showed protons at 1.27, 1.39, 1.53, and 1.75 ppm for the hexyl- and butyl repeating units of monomers present in the polymeric structure. The broad singlets at 3.38 and 4.04 ppm confirmed the presence of neighboring protons of alcohol and ester groups present in the copolymer. Overall, broad peaks observed in the ^1^H NMR spectrum represented the overlap of chemically equivalent protons and characteristics of the synthesized copolymer. In addition, ^13^C NMR spectra were recorded for monomers and HBPEC polymer, as shown in Figure 2. The peak at 171 ppm indicated the presence of ester carbonyl carbons in the backbone, whereas the peak at 60.87 ppm corresponds to the aliphatic carbon chains of 1,6-hexanediol monomeric unit, confirming successful copolymerization. The peak at 64.97 ppm represented the carbon attached to the ester methylene groups, and the peak at 51.94 ppm indicated the presence of DEM’s branching carbon. These spectral features provided information about the structural composition of HBPEC polymer, including the presence of ester groups in the polymeric backbone and alcohol functional groups.

**Figure 3:**
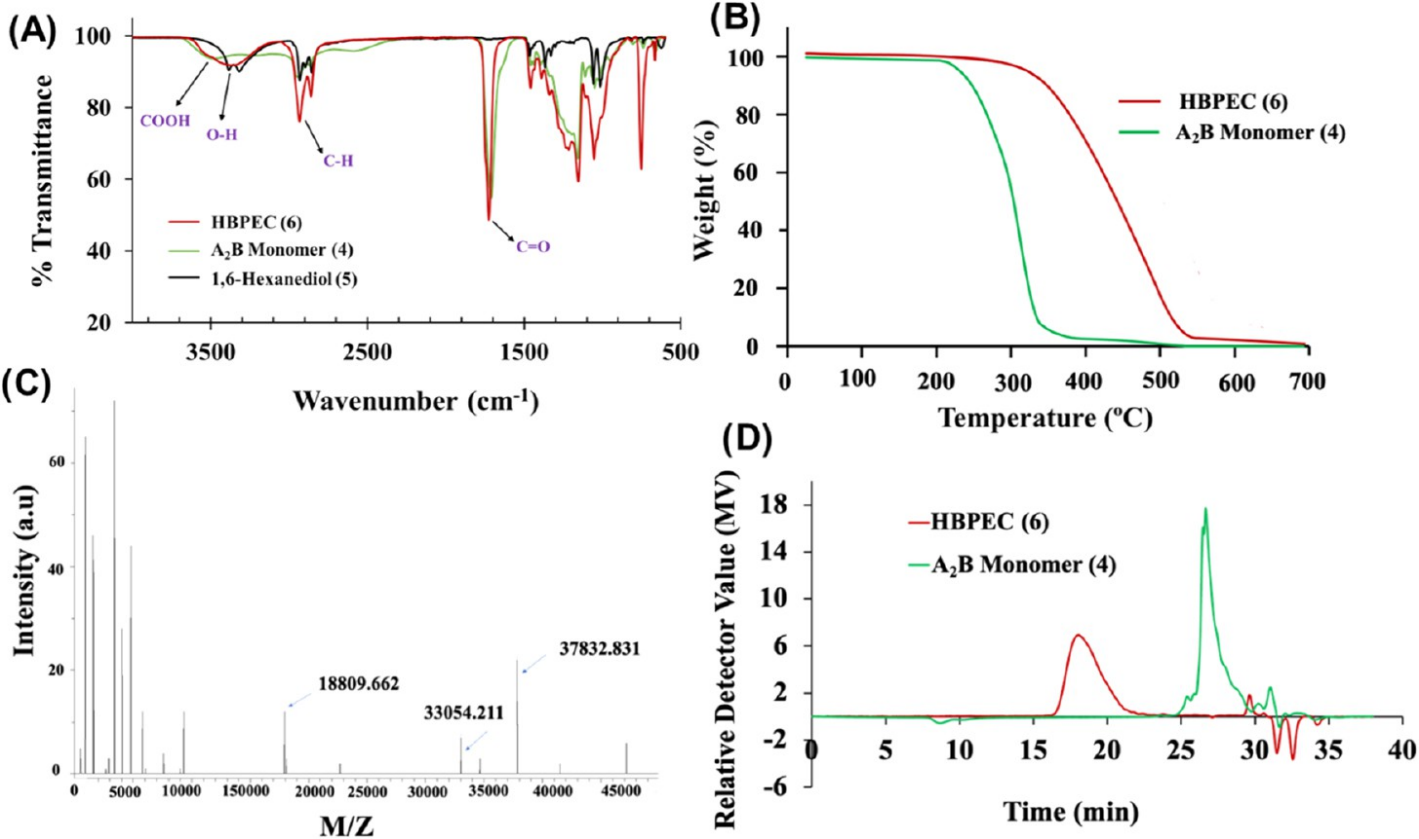
(A) FT-IR, (B) TGA, (C) MALDI-TOF, (D) GPC of the A_2_B monomer (**4**), 1,6-hexanediol monomer (**5**), and HBPEC (**6**).

To further characterize, the FT-IR spectrum of the copolymer was recorded (Figure 3A), which showed a distinct band at 1732 cm^–1^, indicating the presence of ester carbonyl groups in the polymer backbone. It is important to note that the shift of this ester carbonyl band (1732 cm^–1^) from the carboxylic acid carbonyl group (1708 cm^–1^) of the monomer (**4**) further indicated successful polymerization. Thermogravimetric analysis (TGA) of the synthesized monomer and the HBPEC polymer were performed to determine thermal stability. The TGA results (Figure 3B) indicated that the degradation of the polymer’s aliphatic chains with an ester bond was evidenced by a 10% weight loss at 348 °C. This result suggested that the polymer would be amorphous in nature, possibly soluble in common organic solvents, and likely thermodynamically stable at a physiological temperature. The differential scanning calorimetry (DSC) results showed (Supporting Information (SI), Figure S1) that the polymer samples had a glass transition temperature (*T*_g_) of approximately −63 °C. No signs of melting (*T*_m_) or crystallization (*T*_c_) were observed, indicating that the polymer was completely amorphous at room temperature due to the flexibility of the aliphatic polymer backbone with ester linkages. To determine the molecular weight of the synthesized HBPEC polymer, matrix-assisted laser desorption/ionization time-of-flight (MALDI-ToF) experiments were performed (Figure 3C). Results showed a large polymer fragment with an *m*/*z* value of 37,832, indicating the formation of a high molecular weight polymer, which makes it suitable for effective encapsulation of drugs for the targeted delivery. Furthermore, the MALDI-ToF result was complemented by the gel permeation chromatography (GPC) experiments (Figure 3D). The GPC chromatograms of the monomer and HBPEC polymer samples exhibited a larger molecular weight for the polymer sample (*M*_w_ = 47,922; *M*_n_ = 31,528; PDI = 1.52) when compared with that of the monomer. In conclusion, these characterization techniques provided valuable insights into the dendritic structure, thermal stability, molecular weight, and amorphous nature of the synthesized HBPEC polyester copolymer, making it suitable for a drug delivery system.

### 2.2 Formulation of Therapeutic Drug-Encapsulating HBPEC Nanoparticles

**Scheme 2:**
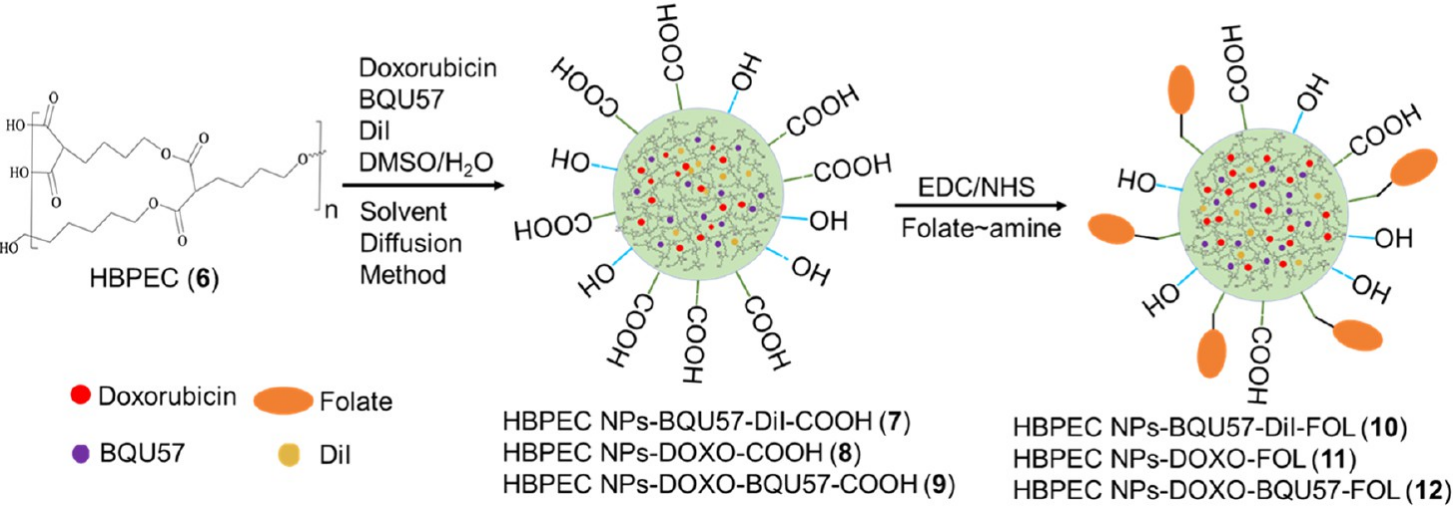
Synthesis of Cargo-Encapsulating, Folate-Decorated HBPEC Nanoparticles

Drug-loaded polymeric nanoparticles were formulated using the solvent diffusion method, as shown in Scheme 2. A solution of HBPEC (30 mg), doxorubicin (5 μL, 2 mg/mL), BQU57 (10 μL, 5 mg/mL), and DiI dye (2 μL, 2 mg/mL) in 250 μL of DMSO was mixed at 1000 rpm for 2 min. The resulting DMSO solution was added dropwise to 4 mL of deionized water under constant stirring, which resulted in the formation of cargo-encapsulating HBPEC nanoparticles (**7**–**9**). The DiI dye was used in the case of BQU57 encapsulating nanoparticle synthesis only (**7**). To target folate-receptor-overexpressing A549 lung cancers, these HBPEC nanoparticles were functionalized with folic acid as the targeting ligand (**10**–**12**). Aminated folic acid was previously synthesized and used for surface conjugation of HBPEC nanoparticles using EDC/NHS carbodiimide chemistry.^68^ The resulting drug-loaded, folate-functionalized nanoparticles were purified *via* dialysis to remove unreacted components. The encapsulation efficiencies (EE) were calculated with the help of spectroscopic methods and using the standard equation, EE% = [(Cargo added–Free cargo)/Cargo added] × 100. The drugs and dyes were incorporated within the polymeric cavities of the dendritic structures with higher encapsulation efficiency (EE_DiI_ = 88%, EE_BQU_ = 82%, EE_Dox_ = 72%).

### 2.3 Characterizations of Functional HBPEC Nanoparticles

**Figure 4:**
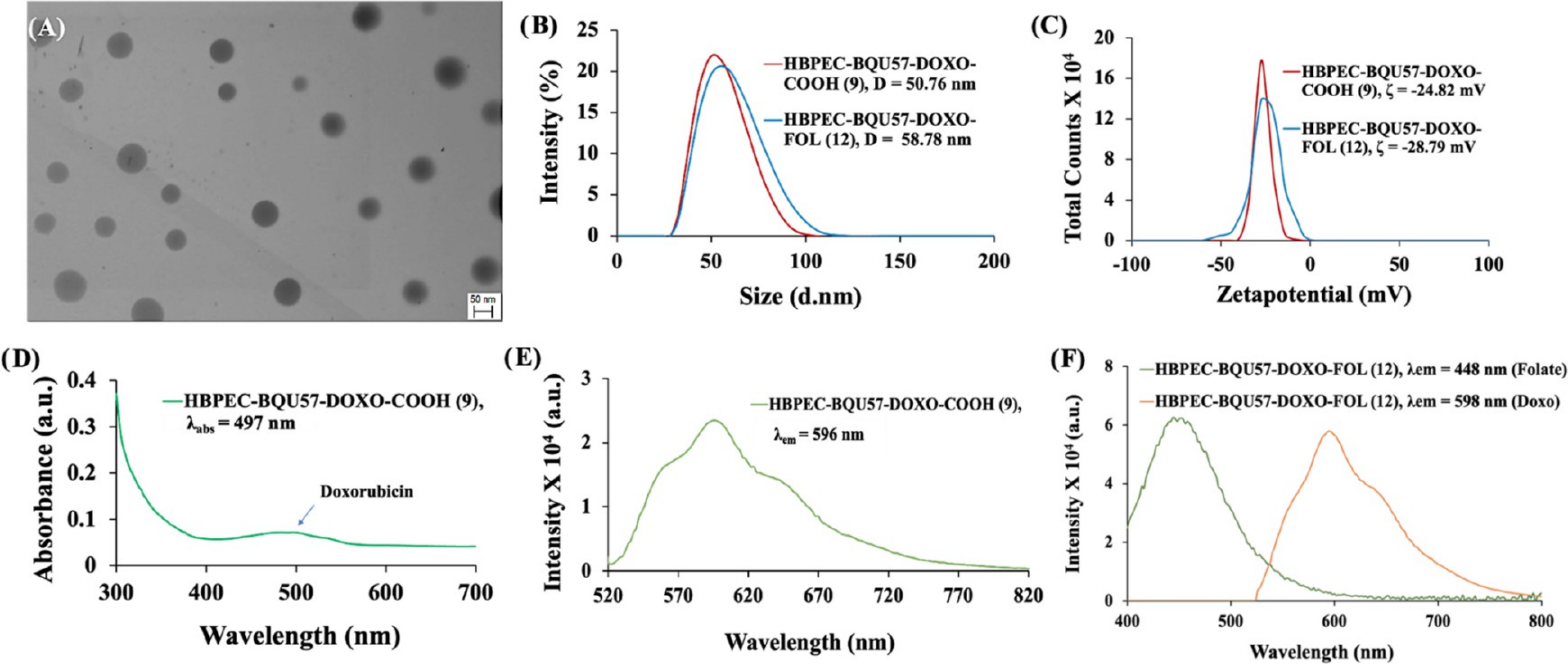
(A) STEM images of nanoparticles (**12**), scale bar: 50 nm. (B,C) Dynamic light scattering (DLS) studies confirmed the presence of stable monodispersed HBPEC–COOH (**9**) and FOL (**12**), and (D–F) encapsulation of doxorubicin was confirmed using spectrophotometric methods and showed fluorescence emission at λ_em_ = 596 nm.

Scanning transmission electron microscopic (STEM) images were collected by depositing nanoparticles (**12**) on a TEM grid, and the acquired images showed the formation of highly dispersed, spherical HBPEC nanoparticles (Figure 4A). In addition, dynamic light scattering (DLS) analysis was performed to measure the hydrodynamic diameter of nanoparticle formulations. The unconjugated HBPEC nanoparticles (**9**) had an average size of approximately 50.76 nm, and the average size was increased to 58.78 nm upon folic acid conjugation (**12**), as illustrated in Figure 4B. The surface charge of the nanoparticles was assessed using ζ-potential analysis and found to be −24.82 mV for carboxylated HBPEC nanoparticles (**9**). After folate conjugation, the nanoparticles showed a ζ-potential of approximately −28.79 mV (Figure 4C), indicating successful surface modification. In addition, ultraviolet–visible (UV–vis) and fluorescence analyses were performed to confirm the successful conjugation of folic acid and encapsulation of doxorubicin and BQU57 within the nanoparticles. The UV/vis absorbance spectrum of carboxylated HBPEC nanoparticles (**9**, Figure 4D) showed a band at λ_abs_ = 497 nm, indicative of the presence of encapsulated doxorubicin drug in solution, which was further confirmed by recording fluorescence emission at λ_em_ = 596 nm (Figure 4E). Distinct fluorescence emission bands were observed at 598 nm for encapsulated doxorubicin and 448 nm for surface-conjugated folic acid when folate-functionalized HBPEC nanoparticles (**12**, Figure 4F) were used. The spectroscopic bands for BQU57 were not visible due to their weak fluorescence properties. However, the BQU57 drug was coencapsulated with the DiI fluorescent dye and folate functionalized to evaluate its therapeutic activity. These HBPEC nanoparticles (**7** and **10**) were characterized using DLS, UV/vis, and fluorescence spectroscopic methods, as shown in the Supporting Information (SI, Figure S2). In summary, these observations validate the successful conjugation of folic acid and encapsulation of doxorubicin and BQU57 drugs within the polymeric cavities of HBPEC nanoparticles. In addition, the preservation of the DiI dye and doxorubicin’s fluorescence properties would greatly enable real-time monitoring of drug delivery and treatment.

### 2.4 *In Vitro* Cytotoxic Studies

**Figure 5:**
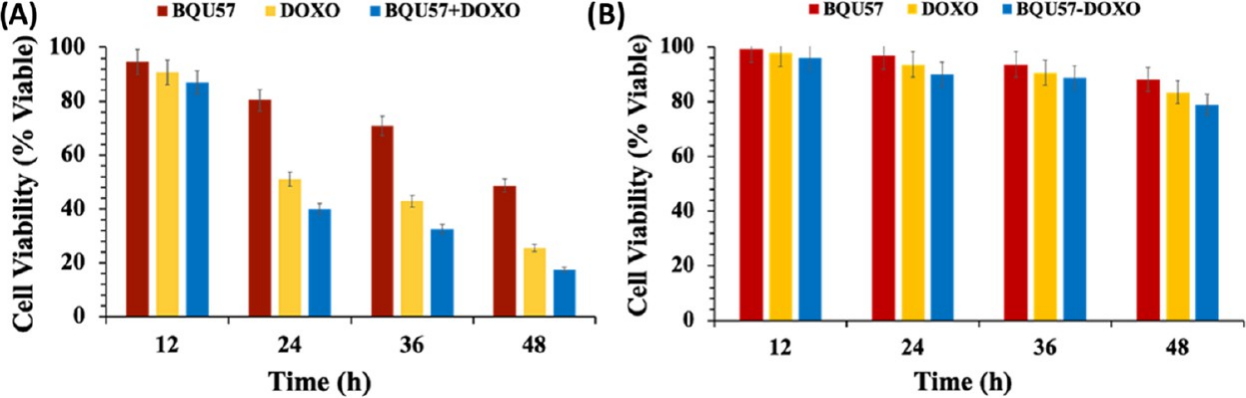
Cell viability experiments using folate-positive A549 cells. (A) Over 82% cell death occurred within 48 h when incubated with HBPEC-folate nanoparticles loaded with both doxorubicin and BQU57 (**12**), whereas 50–70% cytotoxicity was observed when single drug HBPEC-folate nanoparticles were used. (B) Minimal toxicity was observed when nonfolate HBPEC nanoparticles (**7**–**9**) were used due to the lack of cellular internalizations. Experiments were performed in triplicate and calculated against standard error.

To determine the biocompatibility of the HBPEC polymer and to assess the therapeutic efficacy of functional HBPEC nanoparticles, a series of cytotoxicity experiments (MTT assays) were performed in a time-dependent manner using NSCLC (A549, folate-positive) cells and rat cardiomyocyte (H9c2, folate-negative) cells as a control. In these experiments, cells were seeded in a 96-well plate (2500 cells/well) and allowed to grow for 24 h at 37 °C in a 5% CO_2_ incubator. The cells were then incubated with cargo-loaded, nonfolate (**7**–**9**) and folate (**10**–**12**) HBPEC nanoparticles (25 μL, 3 mM) for 12, 24, 36, and 48 h before treating with MTT solution (25 μL, 5 mM) for 4–6 h inside the humidified incubator. The tetrazolium derivative MTT (3-(4,5-dimethylthiazol-2-yl)-2,5-diphenyltetrazolium bromide) reduced to purple-colored formazan in the presence of intracellular mitochondrial dehydrogenase. The therapeutic efficacy of HBPEC nanoparticles was determined by measuring the absorbance intensity of dissolved formazan crystals at 560 nm, where higher intensity signifies better cell viability. The folate-conjugated doxorubicin-HBPEC (**11**) and BQU57-HBPEC (**10**) formulations showed 50 and 20% A549 cell death in 24 h, respectively. However, the folate-HBPEC nanoparticles with a combination of BQU57 and doxorubicin drugs (**12**) showed increased cell death (60%) within 24 h period (Figure 5A). The cell viability was drastically reduced after 48 h of incubation as the combination of drugs (**12**) was highly toxic to the A549 cells and showed over 82% cell death when compared to a single drug, doxorubicin-HBPEC (**11**, 73%) and BQU57-HBPEC (**10**, 50%), nanoparticles. The higher cytotoxicity of the combination therapy indicated that the anticancer properties of doxorubicin were enhanced by the BQU57 drug, maybe due to the two different mechanisms of action. The nonfolate HBPEC nanoparticles (**7**–**9**) showed a minimal reduction in cell viability, approximately 20% cell death in 48 h (Figure 5B), due to the lack of folate receptor-mediated internalizations. In addition, when these HBPEC nanoparticles were incubated with folate receptor-negative H9c2 cells, substantially lower cell death was observed (SI, Figure S3). This is due to the lack of HBPEC nanoparticles’ internalizations, shown by less than 25% cell death when incubated with HBPEC-folate nanoparticles (**10**–**12**) and 15% cytotoxicity for nonfolate formulations (**7**–**9**). These results indicated that the HBPEC nanoparticles (**12**) were degraded upon internalizations, released drugs, and initiated apoptosis synergistically by initiating two different pathways. In summary, the HBPEC-folate nanoparticles showed high selectivity to A549 cells while minimizing its cytotoxicity to healthy cells. In addition, the combination of drug selection would bring a potential solution in overcoming multidrug resistance (MDR) and treating undruggable NSCLC.

### 2.5 Cellular Internalization

**Figure 6:**
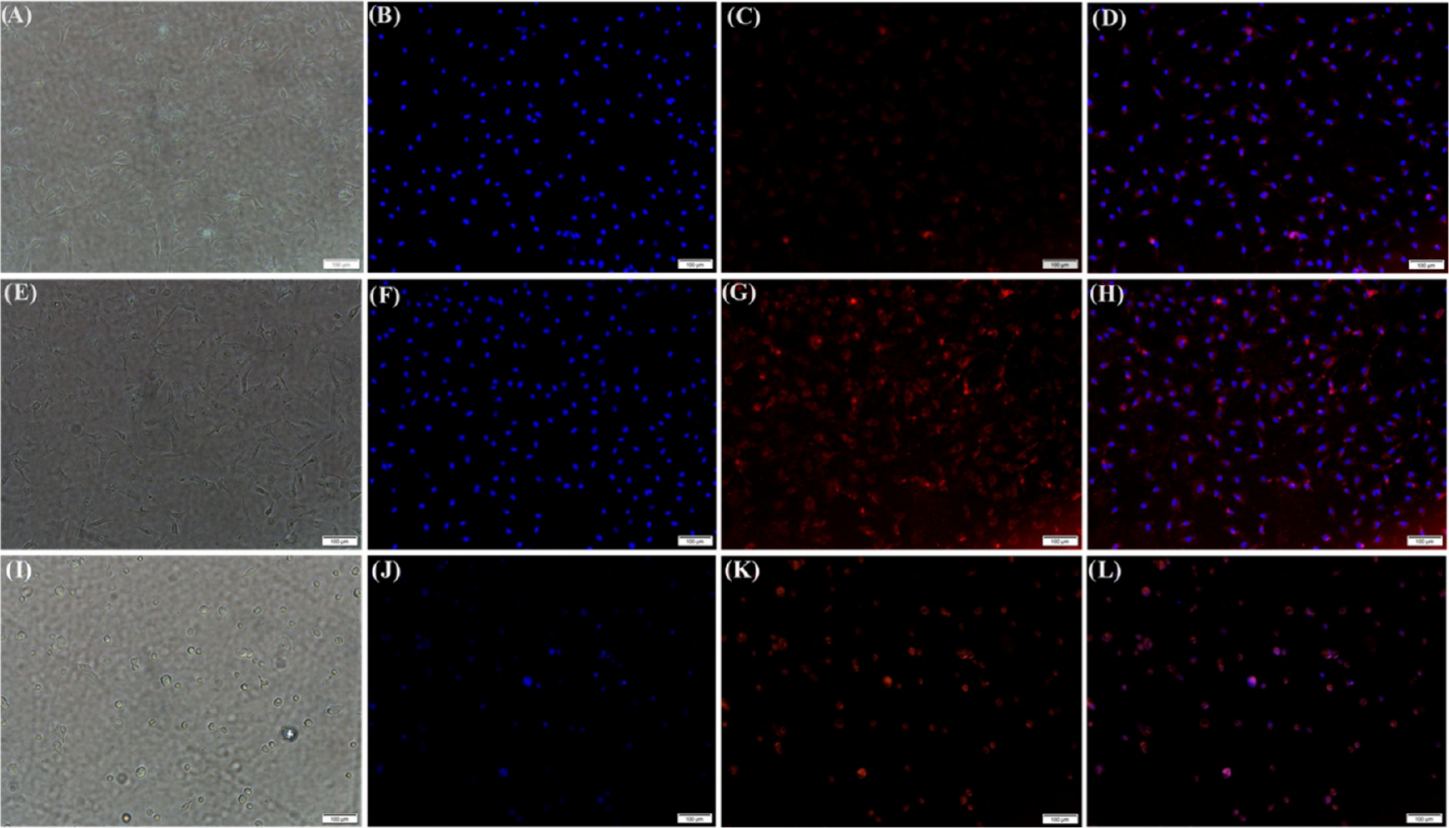
Representative fluorescence microscopic images (scale bar 100 μm) for *in vitro* cellular imaging, nuclei were stained with DAPI dye (blue). (A–D) Minimal internalization of HBPEC-BQU57-DOXO–COOH nanoparticles (**9**) was observed in A549 cells (scale bar 100 μm). (E–H) Cellular internalization of HBPEC-BQU57-DOXO-FOL (**12**) was observed due to the folate-receptor-mediated endocytosis, and (I–L) cell death was observed within 48 h when HBPEC-BQU57-DOXO-FOL (**12**) was incubated with A549 cells.

Fluorescence microscopy was utilized to monitor the intracellular uptake of HBPEC nanoparticles and to assess their therapeutic efficacy. A549 cells were incubated with nonfolate (**9**) and folate-HBPEC nanoparticles (**12**, 50 μL, 3 mM) for 24 h before fixing the cells with 4% paraformaldehyde. Another experiment was set for 48 h using folate-HBPEC nanoparticles (**12**, 50 μL, 3 mM) to visualize the results of therapeutic treatment. The DAPI dye was used to stain the cell nuclei, resulting in blue-colored fluorescence, while the internalizations were tracked by doxorubicin’s red fluorescence emission at 498 nm. When nonfolate HBPEC nanoparticles (**9**, 50 μL, 3 mM) were incubated for 24 h, minimal internalization was observed (A–D, Figure 6) due to the lack of folate ligands and no effective internalizations. On the other hand, successful internalizations were observed within 24 h in the case of folate-conjugated HBPEC-BQU57-DiI (A–D, SI Figure S4) and HBPEC-BQU57-DOXO nanoparticles (E–H, Figure 6). However, when this incubation was extended over 48 h, a significant amount of mitotic cell arrest, leading to cell death, was observed (I–L, Figure 6). These results demonstrate the effective receptor targeting and drug delivery to cancer cells. Additionally, these results corroborate the MTT assay for cell viability and further indicate that the drug selections in this combination therapy would reduce drugs’ cardiotoxicity and MDR for an effective NSCLC treatment option.

### 2.6 Detection of Intracellular Reactive Oxygen Species

**Figure 7:**
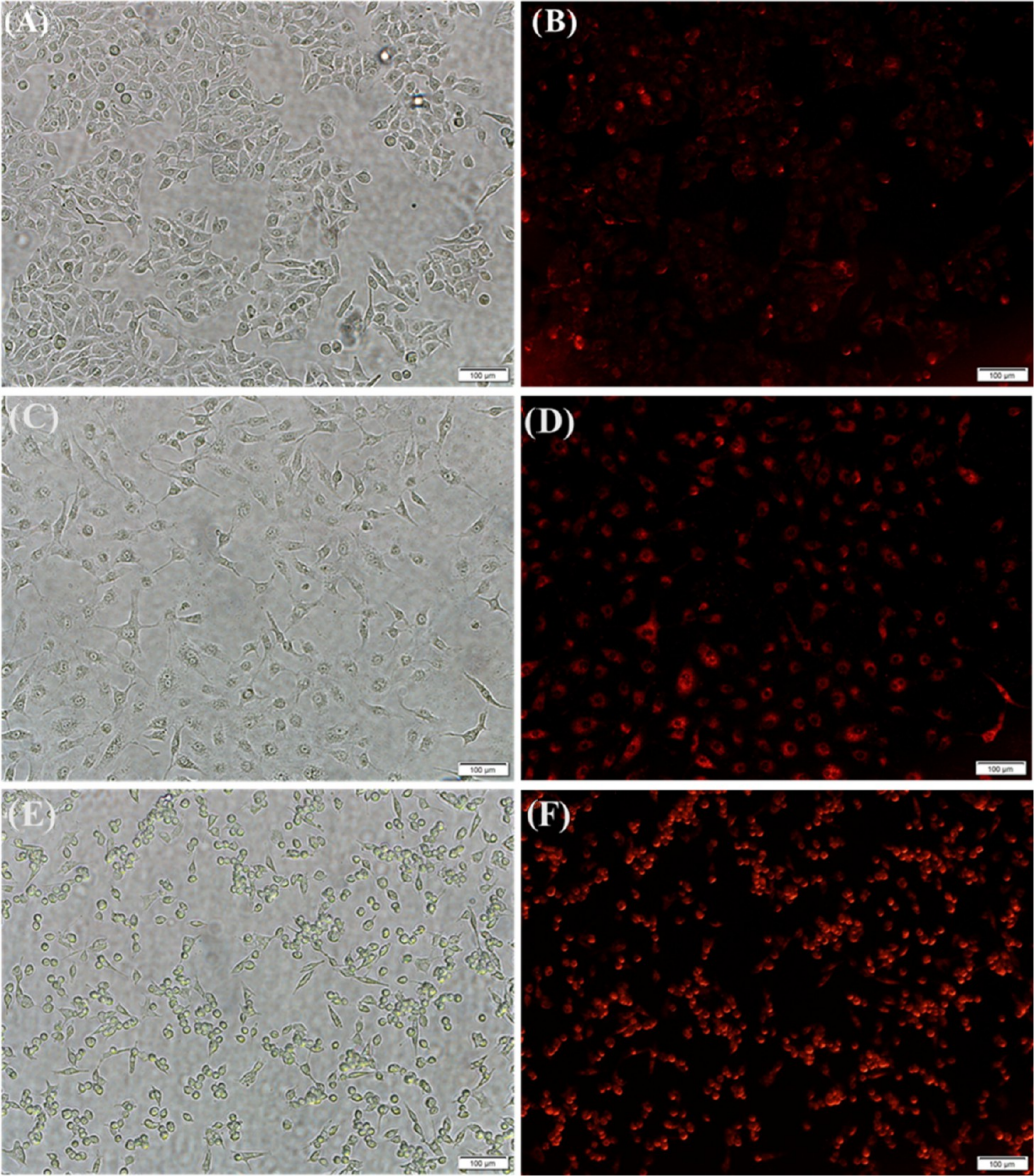
Determination of ROS in the A549 cell line (scale bar 100 μm). Representative microscopic images of the generation of cytoplasmic ROS in the presence of (A, B) HBPEC-BQU57-DOXO–COOH (**9**), (C,D) HBPEC-DOXO-FOL (**11**), and (E, F) HBPEC-BQU57-DOXO-FOL (**12**), which are labeled with the DHE dye.

A growing body of evidence suggests that intracellular levels of reactive oxygen species (ROS) are crucial for the maintenance of redox-cellular homeostasis, regulation of various biological functions, and potentiation of programmed cancer cell death through the induction of oxidative stress. To determine whether HBPEC-BQU57-DOXO-FOL nanoparticle-induced cytotoxicity in A549 cancer cells is triggered due to elevated ROS levels, a cell-based assay was performed using a fluorescent probe dihydroethidium (DHE). DHE, a blue color fluorescent dye, is oxidized to 2-hydroxyethidium in the presence of superoxides and hydrogen peroxide and hence can be used for the direct detection of intracellular ROS in live cells. Briefly, A549 cells were grown in small Petri dishes and treated with different nanoparticles: carboxylated nanoparticles (**9**), folate-functionalized nanoparticles (**10**, **12**), and H_2_O_2_ (as a positive control, results not shown) for 6 h. The cells were then stained with the DHE dye (32 μM) for 30 min and fixed with 4% paraformaldehyde. The results showed that carboxylated nanoparticles (**9**) exhibited minimal red fluorescence, indicating low ROS generation (Figure 7A,B). As shown in Supporting Information Figure S5, folate-functionalized nanoparticles with BQU57 therapeutic drug (**10**) showed some extent of red fluorescence, suggesting a modest increase in ROS levels. However, folate-HBPEC nanoparticles with doxorubicin (**11**) demonstrated a relatively higher red fluorescence, indicating an increased ROS generation (Figure 7C,D). Doxorubicin, an anthracycline-based anticancer drug, is known to exert its effect through the chelation of intracellular iron, leading to elevated levels of hydroxyl radicals and eventually programmed cell death. To determine whether BQU57 promotes the therapeutic efficacy of doxorubicin by directly enhancing its ROS accumulating activity, the cells were incubated with the folate nanoparticles (**12**) containing a combination of drugs for 6 h. The results are shown in Figure 7E,F with an increased level of red fluorescence, suggesting further ROS elevation. Based on these results, it was evident that the generation of ROS might be one of the central mechanisms responsible for the superior cytotoxic activity of the HBPEC-BQU57-DOXO-FOL nanoparticle in A549 lung cancer cells.

### 2.7 Determination of Apoptotic and Necrotic Pathways

**Figure 8:**
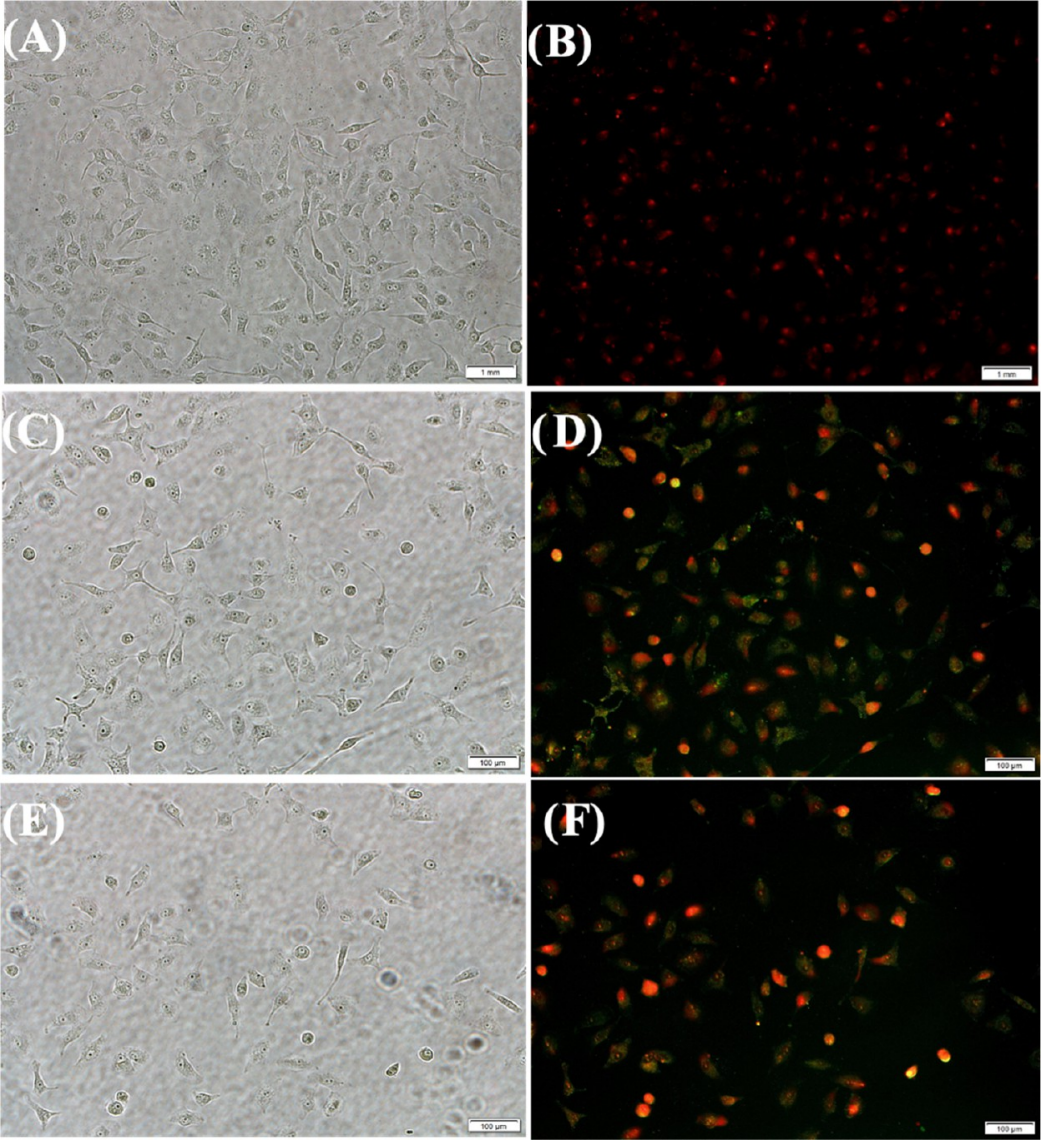
Determination of apoptosis and necrosis events in A549 cells after treatment with (A, B) HBPEC-BQU57-DOXO–COOH (**9**), (C,D) HBPEC-DOXO-FOL (**11**), and (E, F) HBPEC-BQU57-DOXO-FOL (**12**) for 24 h (scale bar 100 μm). A549 cells were then stained with Annexin V-FITC and ethidium homodimer III dyes.

Acceleration of accumulative ROS beyond a certain threshold in cancer cells has been shown to result in their selective killing *via* the activation of several cellular processes, including apoptosis, autophagic cell death, and necroptosis. Experiments were conducted to examine whether ROS induction in the presence of HBPEC-BQU57-DOXO-FOL nanoparticle (**12**) triggered an apoptotic signaling pathway. In these experiments, cells were grown in small Petri dishes for 24 h and then treated with carboxylated nanoparticles (**9**, 25 μL, 3 mM) and folate-functionalized nanoparticles (**12**, 25 μL, 3 mM) for 24 h. Following the incubations, cells were subjected to staining with fluorescent dyes Annexin V-FITC and Ethidium homodimer III to detect apoptotic and necrotic pathways of cell death. Apoptotic and necrotic cells were distinguished based on their morphology and differential staining, as they exhibited green and red fluorescence, respectively. As shown in Figure 8A,B, the control experiments using carboxylated HBPEC-BQU57-DOXO–COOH nanoparticles (**9**) did not result in any detectable apoptotic and necrotic cells, reflecting the lack of any effective internalizations of the nonfolate nanoparticles. In addition, folate-HBPEC nanoparticles with doxorubicin (**11**, Figure 8C,D) and BQU57 drug (**10**, Figure S6) resulted in early apoptosis, as indicated by the presence of major green spots (representing apoptosis) and fewer red spots (representing necrosis). However, incubation of HBPEC-BQU57-DOXO-FOL nanoparticle (**12**, Figure 8E,F) resulted in an increase in necrotic cells. Overall, these results indicate that doxorubicin and BQU57-loaded HBPEC nanoparticles triggered apoptotic and necrotic signaling pathways in A549 cells and could provide an efficient strategy for combination therapy in NSCLC.

### 2.8 Comet Assay

**Figure 9:**
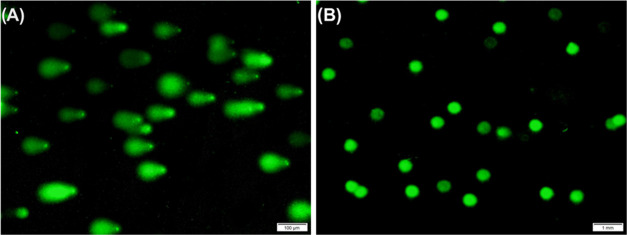
Comet assays were performed on the A549 cells using functional HBPEC nanoparticles. Representative microscopic images of the comet assay (A) in the presence of HBPEC-BQU57-DOXO-FOL (**12**) nanoparticles showing the formation of long comet tails. (B) Control comet experiment in the presence of HBPEC-BQU57-DOXO–COOH nanoparticles (**9**) with no visible comet formations.

To establish the mechanism of therapeutic efficacy of HBPEC nanoparticles containing doxorubicin and BQU57 against A5549 cancer cells, an alkaline comet assay was performed. Doxorubicin, a chemotherapeutic drug, is known to exert its effect by intercalating with DNA bases and thus breaking DNA strands. Figure 9A,B shows the representative microscopic images of the comet experiments conducted in the presence of folate-functional and carboxylated HBPEC nanoparticles. DNA damage was observed to be significant upon treatment with HBPEC-BQU57-DOXO-FOL (**12**, Figure 9A), as indicated by the presence of intense and long comet tails. In addition, similar comet tails were captured when treated with HBPEC-DOXO-FOL nanoparticles (**11**), and as expected, no substantial comet was observed when HBPEC-BQU57-DiI-FOL nanoparticles (**10**) were used (SI, Figure S7). Based on these observations, it is evident that the formulated HBPEC nanoparticles can successfully deliver combination drugs to the cytoplasm, which is then translated to the nucleus. However, carboxylated nanoparticle (**9**) treatment resulted in minimal DNA damage, as observed by the absence of any comet tails (Figure 9B). Taken together, these results suggest the efficacy of the newly formulated HBPEC nanoparticles as a drug delivery platform.

### 2.9 Migration Assay

**Figure 10:**
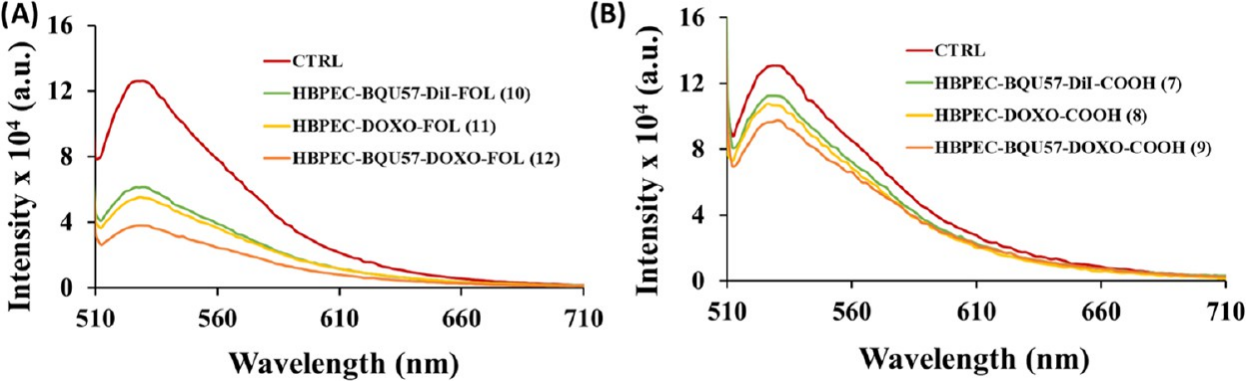
Determination of the antimetastatic effect of the combination therapy using the migration assay. Control experiments (CTRL) used no HBPEC nanoparticles. (A) The migration assay using folate-conjugated HBPEC nanoparticles (**10**–**12**) showed decreased migration of A549 cells. (B) Nonfolate HBPEC nanoparticles (**7**–**9**) showed a minimal migration inhibitory effect.

Metastases of NSCLC continue to be a common complication during the treatment and a hallmark event of such cancers. To assess whether HBPEC-BQU57-DOXO-FOL nanoparticles (**12**) can inhibit the invasion and migratory abilities of A549 cells, a transwell migration assay was performed. The assay involved growing cells in serum-free media and treating them with carboxylated and folate-functionalized HBPEC nanoparticles (**7**–**12**, 25 μL, 3 mM). The invasion chamber was filled with serum-free media, and the setup was incubated for 24 h. Following the Chemi-Con protocols for the assay, the fluorescent dye CYQUANT was used to measure the fluorescence intensity. As shown in Figure 10, A549 cancer cells without any treatment with HBPEC nanoparticles (CTRL: control experiment) migrated from the invasion chamber to the lower feeder tray and exhibited higher fluorescence intensity. However, there was a significant reduction in fluorescence intensity upon treatment with folate-conjugated HBPEC nanoparticles (**10**–**12**, Figure 10A), indicating a decrease in the migration ability of A549 cancer cells. On the contrary, carboxylated HBPEC nanoparticles (**7**–**9**) showed similar fluorescence as observed from control cells, indicating a minimal effect on cell migration (Figure 10B). Collectively, the obtained results revealed that folate-decorated HBPEC nanoparticles containing both drugs had higher potential in inhibiting the invasion and migratory abilities than the folate nanoparticles containing single drugs. These results demonstrated that our functional HBPEC delivery system with the combination of drugs has the potential to prevent NSCLC metastasis effectively.

## 3 Experimental Section

### 3.1 Materials

Ethylenediamine (EDA), *N*-hydroxysuccinimide (NHS), and 1,6-hexanediol were obtained from Sigma-Aldrich. *N*–*N*′-Dimethyl sulfoxide, *p*-toluenesulfonic acid (*p*TSA), 3-(4,5-dimethylthiazol-2-yl)-2,5-diphenyltetrazolium bromide (MTT), 1-ethyl-3-[3-dimethyl- aminopropyl]carbodiimide hydrochloride (EDC), doxorubicin, trifluoroacetic acid (TFA), and regular solvents, including tetrahydrofuran (THF), hexane, and ethyl acetate, were purchased from Fisher Scientific and used as received. Deuterated NMR solvents dimethyl sulfoxide (DMSO-*d*_6_) and chloroform (CDCl_3_) were purchased from Cambridge Isotope Laboratories. The near-infrared dye (DiI-D282), dihydroethidium (DHE), and 4′,6-diamidino-2-phenylindole (DAPI) were purchased from Invitrogen. The human lung carcinoma cell lines A549 (CCL-185) and cardiomyocytes (H9c2) were obtained from the American Type Culture Collection (ATCC). DMEM media, fetal bovine serum, and antibiotics were obtained from Corning. The dialysis membrane (MWCO = 6–8 K) was purchased from Spectrum Laboratories.

### 3.2 Instrumentations

#### 3.2.1 FT-IR (Fourier Transform-Infrared) Spectroscopy

The A_2_B monomer, 1,6-hexanediol, and HBPE copolymer neat samples were analyzed using a PerkinElmer Spectrum 2 FT-IR spectrometer.

#### 3.2.2 ^1^H and ^13^C NMR (Nuclear Magnetic Resonance) Spectroscopy

The copolymer (40 mg) and monomers (20 mg) were dried under a vacuum and dissolved in DMSO-*d*_6_. The samples were then analyzed using a Bruker DPX-300 MHz spectrometer with the TOPSPIN 1.3 program.

#### 3.2.3 Gel Permeation Chromatography (GPC) and Matrix-Assisted Laser Desorption/Ionization-Time of Flight (MALDI-ToF)

The copolymer and monomers (20 mg/mL) were dissolved in THF and passed through a 0.2 μm filter. The samples were then subjected to GPC analysis using a Waters 2410 DRI gel permeation chromatograph with polystyrene-divinylbenzene beads-loaded columns. The THF solvent flow rate was kept at 1 mL/min at 25 °C for 50 min. Bruker microflex LRF MALDI-ToF was used for the analysis of the HBPEC polymer sample. The matrix solvent was prepared using 2 mg of dihydroxybenzoic acid (DHB) in 100 μL of TA30 solvent (30:70 volume ratio of acetonitrile in DI water to 0.1% trifluoroacetic acid) and used for 2 mg of vacuum-dried HBPEC polymer sample, vortexed and spotted on a MALDI target plate.

#### 3.2.4 Thermogravimetric Analysis (TGA)

The thermal stability of the copolymer was investigated using a TGA Q50 Thermogravimetric analyzer from TA Instruments. The samples (10 mg) were heated under a N_2_ gas environment at a ramp rate of 10 °C/min, ranging from 25–700 °C. TGA provides information about the thermal decomposition behavior of the samples.

#### 3.2.5 Dynamic Light Scattering (DLS) and Scanning Electron Microscopy (SEM)

The nanoparticle formulations were analyzed using Malvern’s ZS90 Zetasizer with approximately 100 readings in 3 cycles. The samples (10 μL/mL, 3 mM) were placed in appropriate cuvettes (Malvern DTS1070 folded capillary cells for ζ-potential measurements) for DLS measurements. Scanning Electron Microscopic images were acquired using JEOL 7600F in an STEM mode.

#### 3.2.6 Absorbance and Fluorescence Measurements

The spectroscopic measurements of the nanoparticle formulations were measured using a Tecan Infinite M200 Pro microplate reader. The samples (100 μL/mL, 3 mM) were placed in a 96-well plate, and the absorbance and fluorescence emission measurements were recorded over a range of wavelengths.

### 3.3 Synthesis and Characterizations

#### 3.3.1 2-(4-Hydroxybutyl)malonic Acid (A_2_B Monomer)

The diacid A_2_B monomer (**4**, Scheme 1) was synthesized using a two-step method as previously reported.^62,63^ Briefly, the compound 2-(4-acetoxybutyl)malonic acid diethyl ester (**3**) was dissolved in methanol and NaOH solution (5:1) and heated to 90 °C for 8 h. After completion of the reaction, the reaction mixture was cooled to room temperature and acidified using diluted HCl to pH 3. The mixed solvent was removed using a rotary evaporator under reduced pressure. To remove any excess hydrochloric acid, Argon gas was continuously bubbled through the CHCl_3_ solution of the reaction mixture at 60 °C. The mixture was filtered to remove any solid impurities and concentrated. Purification of the product (**4**) was achieved using column chromatography with a 35% ethyl acetate in petroleum ether mixture as the eluent.

**Yield**: 2.30 g (71%). ^**1**^**H NMR** (300 MHz, DMSO-*d*_6_, δ ppm, *J* Hz): 1.42 (m, 2H), 1.58 (m, 2H), 1.92 (q, 2H, *J*_1_ = 7.4, *J*_2_ = 7.7), 3.36 (t, 1H, *J* = 7.3), 3.65 (t, 2H, *J* = 6.4), 5.53 (bs, 1H). ^**13**^**C NMR** (75 MHz, DMSO-*d*_6_, δ ppm): 23.68, 28.46, 32.68, 51.94, 60.87, 170.79. **FT-IR** (CHCl_3_): 3510, 2955, 1708, 1626, 1459, 1438, 1396, 1198, 1161, 1052, 947, 772, 743, 667 cm^–1^. **TGA**: 10% weight loss at 268 °C.

#### 3.3.2 2-(4-Hydroxybutyl)malonic Acid-Hexanediol Hyperbranched Polyester Copolymer (HBPEC)

The purified A_2_B monomer (**4**) and 1,6-hexanediol (**5**, 1:1 equiv) were added in a 5 mL round-bottom flask (RBF), mixed, and purged with ultrapure nitrogen gas to create an inert atmosphere. The catalyst *p*-toluenesulfonic acid (*p*TSA, 100:1 ratio) was added to the round-bottom flask and heated to 140 °C for 2 h. The process was carried out under a medium vacuum (1.5 mmHg) for 8 h, and the resulting copolymer was dissolved in dimethyl sulfoxide (DMSO). This polymer solution was purified using mixed-solvent precipitation from deionized water and centrifuged. The polymer was then dried at 40 °C in a vacuum oven overnight. The obtained HBPEC was found to be a clear viscous liquid and readily soluble in chloroform, DMSO, and tetrahydrofuran. The obtained pure polymer (**6**, Scheme 1) was characterized using NMR, FT-IR, GPC, TGA, and MALDI-ToF to ensure purity, structure, and molecular weight.

**Yield**: 51%. ^**1**^**H NMR** (300 MHz, DMSO-*d*_6_, δ ppm): 1.27 (bs, 6H), 1.39 (m, 2H), 1.53 (bs, 4H), 1.75 (m, 2H), 2.49 (bs, 1H), 3.38 (m, 4H), 3.41 (bs, 1H), 4.04 (bs, 2H). ^**13**^**C NMR** (75 MHz, DMSO-*d*_6_, δ ppm): 23.68, 28.69, 32.68, 51.94, 60.87, 64.97, 170.94, 171.43. **FT-IR**: 3408, 2935, 2863, 1732, 1462, 1391, 1234, 1162, 1104, 1056, 752, 666 cm^–1^.**TGA**: 10% weight loss at 348 °C.

#### 3.3.3 Drug-Encapsulating HBPEC Nanoparticle (**7**–**9**) Formulation

Therapeutic drugs and optical dye encapsulating HBPEC nanoparticles were formulated using the solvent diffusion method.^63^ Typically, to a DMSO solution (500 μL) of HBPEC (**6**, 30 mg) either [BQU57 (4 mM) + DiI (2 mM)] (**7**), [Doxorubicin (Doxo) (4 mM)] (**8**), or [BQU57 (4 mM) + Doxo (4 mM)] (**9**) were mixed thoroughly and slowly added dropwise to a 15 mL Eppendorf tube containing 4.0 mL DI water with continuous vortexing at 1000 rpm (Scheme 2). The cargo-encapsulating nanoparticles were purified by dialysis for 3 h using a dialysis membrane with a molecular weight cutoff (MWCO) of 6–8 kDa. This step helped to remove any unencapsulated cargo and solvents from the nanoparticles. After dialysis, the resulting solution (5 mM) appeared milky and was found to be stable at room temperature. The resulting formulation was stored at 4 °C until further use.

#### 3.3.4 Folate-Functionalized Drug-Encapsulated HBPEC Nanoparticles (**10**–**12**)

Aminated folic acid was previously synthesized using folic acid and ethylenediamine by following EDC/NHS chemistry.^63,69,70^ This was then used to folate-functionalize our nanoparticles (**7**–**9**) using the standard EDC/NHS chemistry. In this process, three different solutions were prepared: Solution 1 [EDC (10 mM) dissolved in 100 μL of MES buffer pH 6.0] was added to carboxylated nanoparticles (**7**–**9**, 5 mM), incubated for 3 min before adding Solution 2 [NHS (10 mM) dissolved in 100 μL of MES buffer] and incubated for 5 min. Finally, Solution 3 [folate amine (10 mM) dissolved in 200 μL of phosphate-buffered saline (PBS) (pH 7.4)] was added dropwise to the resulting nanoparticles and incubated for 3 h on a tabletop mixer at room temperature. The functionalized nanoparticles (**10**–**12**) were dialyzed overnight using a dialysis membrane with a molecular weight cutoff (MWCO) of 6–8 kDa. The purified nanoparticles (3 mM) were stored at 4 °C for characterization and subsequent experiments.

### 3.4 *In Vitro* Cell Culture Studies

The lung cancer cells (A549) and rat cardiomyocytes (H9c2) were grown in an incubator (37 °C and 5% CO_2_) using vacuum-filtered, sterilized DMEM media supplemented with 10% fetal bovine serum and 1% streptomycin antibiotic.

#### 3.4.1 MTT Assay

The H9c2 and A549 cells were seeded in a 96-well plate and allowed to grow for 24 h to reach ∼2500 cells per well. The functional HBPEC nanoparticles (**7**–**12**, 25 μL, 3 mM) were added to each well containing the respective cell lines and incubated for 12–48 h. After the incubation period, the media in each well was removed, and cells were washed 3 times with 1× PBS. Next, 25 μL of MTT (5 mg/mL) solution was added to each well and incubated for 4–6 h to allow the cells to metabolically reduce the MTT dye. Afterward, the purple-colored formazan crystals were formed and dissolved in acidic (0.1 N HCl) isopropanol. The absorbance of the solution was measured at 560 nm using a Tecan Infinite M200 Pro multidetection microplate reader. The absorbance measurement provides an indication of cell viability, as the intensity of the color is directly proportional to the number of viable cells. The experiments were repeated 3 times for reproducibility.

#### 3.4.2 Cell Internalization Studies

For the internalization experiments, A549 cells were seeded in Petri dishes and allowed to culture for 24 h (cell count: 10,000 cells/dish). HBPEC nanoparticles (**9**, **12**, 50 μL, 3 mM) were added to the cells and incubated for 24–48 h for effective cellular internalizations. After incubation, the dishes were washed 3 times with 1× PBS (pH 7.4) and fixed using 4% paraformaldehyde. Fixation helps preserve the cellular structure and prevents cell detachment during subsequent DAPI staining (25 μL, 1.0 μM) of cell nuclei and microscopy using a fluorescence microscope (Olympus 1 × 73) equipped with appropriate filters for DAPI staining.

#### 3.4.3 Determination of Cytosolic ROS Stress

Lung cancer cells were used to assess the generation of reactive oxygen species (ROS) upon treatment with functional nanoparticles. The A549 lung cancer cells were seeded in various Petri dishes and allowed to grow for 24 h (cell count: 10,000 cells/dish). Nanoparticles (**9**, **10**, **12**; 25 μL, 3 mM) were incubated for 24 h before staining with the dihydroethidium (DHE, 32 μM) dye for 30 min. The DHE dye is used to detect and quantify intracellular ROS levels, whereupon oxidation in the presence of ROS, it fluoresces red. The stained cells were observed under a fluorescence microscope (Olympus 1 × 73) equipped with appropriate filters to visualize the red fluorescence emitted by the oxidized DHE dye.

#### 3.4.4 Apoptosis and Necrosis Assay

The A549 cells were seeded in two separate Petri dishes and allowed to grow for 24 h (10,000 cells/dish). Nanoparticles (**9**, **10**, **12**; 25 μL, 3 mM) were incubated for 24 h and then treated with 4% paraformaldehyde for cell fixation. After washing 3 times with 1× PBS, cells were stained with FITC (5 μL) and ethidium homodimer (III) dyes (3 μL) for 15 min using a kit from Biotium. FITC is a fluorescent green dye used to label apoptotic cell components, while the ethidium homodimer (III) red dye is used to assess necrotic cells with compromised membranes. The stained cells were then washed with 1× Annexin binding buffer. The images were taken using an Olympus inverted fluorescence microscope equipped with appropriate filters.

#### 3.4.5 Comet Assay

Comet assays were performed using A549 cells to assess DNA damage. A549 cells were seeded in two different Petri plates with 10,000 cells per plate and allowed to grow for 24 h. Nanoparticles (**7**, **9**, **12**; 25 μL, 3 mM) were added and incubated for an additional 24 h. After incubation, the cells were trypsinized using 2% trypsin solution, detached from the plates, and collected by centrifugation at 1000 rpm for 6 min. The resulting cell pellet was resuspended in 1× PBS (500 μL) solution. The cell suspension was mixed with preheated agarose gel at 37 °C, spread on a comet slide, and allowed to solidify for 30 min in a dark environment at 4 °C. After solidification, the slides with the agarose gel were immersed in a cell lysis solution for 30 min. This step helped to remove cellular proteins and membranes, leaving behind the DNA. The slides were then immersed in an alkaline unwinding solution with a pH greater than 13 for 1 h. This step promotes DNA unwinding and denaturation. The slides were transferred to an electrophoresis solution with a pH greater than 13 and subjected to electrophoresis for 30 min at 21 V. This step causes the fragmented DNA strands to migrate, forming a comet-like shape. After electrophoresis, the slides were rinsed twice with DI water and then with 70% ethanol for 5 min each. The gel was then stained with the fluorescent SYBR Gold dye (100 μL) for 30 min at room temperature to bind with DNA. Following staining, the slides were dried for 15 min at 37 °C, and the images were taken using an Olympus fluorescence microscope equipped with a FITC filter.

#### 3.4.6 Transwell Migration Assay

The Millipore-Sigma migration kit has a cell invasion chamber (insert), a 96-well feeder tray, and a base for performing migration assay. The insert contains an extracellular matrix (ECM) that mimics the physiological environment for cell invasion. The A549 cells were incubated in the inset for 1–2 h with prewarmed serum-free media. This step allows the ECM to rehydrate and provides a suitable environment for cell invasion. Next, the media in the inset was carefully removed without disturbing the ECM, and 150 μL of serum-free media was added to the feeder tray to create a chemo-attractant gradient for cell migration. The A549 cells (100 μL, 4000 cells) were added to each well of the inset. The HBPEC nanoparticles (**9**, **12**; 1–10 mM) were added to the respective wells and incubated for 24 h for migration. The insert was washed with PBS to remove noninvading cells, and 150 μL of prewarmed cell detachment solution was added and then incubated for 30 min. In the feeder tray, 50 μL of CYQUANT dye and 4× lysis buffer (1:4 ratio) were added and incubated at room temperature for 15 min. A fluorescence emission scan was performed at 520 nm using a Tecan Infinite M200 multidetection microplate reader.

## 4 Conclusions

In conclusion, we have synthesized a new HBPEC polymer for effective encapsulation and delivery of hydrophobic anticancer drugs within the polymeric cavities. Using the A_2_B monomer and 1,6-hexanediol, the HBPEC polymer was synthesized that exhibited excellent properties, including higher molecular weight, better solubility, and thermostability. Further, the presence of carboxylic acid functionality on the polymer surface allowed modifications and facile conjugation of targeting ligands, whereas the aliphatic ester linkages in the polymeric backbone made it biocompatible. Following polymer characterizations, a one-pot solvent diffusion method was used, which resulted in the formation of HBPEC nanoparticles with higher encapsulation efficiencies for anticancer drugs doxorubicin and BQU57. To facilitate targeted delivery of therapeutic drugs to A549 cancer cells, the surface of HBPEC nanoparticles was covalently linked with folic acid using EDC/NHS carbodiimide chemistry. Doxorubicin and BQU57-loaded HBPEC nanoparticles showed potent anticancer activity as indicated by efficient cellular internalization and remarkable cellular cytotoxicity in A549 cancer cells with 80% cell death after 48 h of incubation. However, no toxicity was observed in healthy H9c2 cells. Further, the mechanism of cytotoxicity of these drug-loaded HBPEC nanoparticles in cancer cells was confirmed by several cell-based assays, including ROS, apoptosis, and comet experiments. These therapeutic nanoplatforms also demonstrated antimetastatic activity based on the results obtained from the transwell migration assay performed on A549 cancer cells. Overall, our findings underline the potential of our newly synthesized HBPEC polymer as a promising platform for the targeted delivery of hydrophobic anticancer drugs and warrants further investigation as a drug delivery platform for the treatment of other cancers.

## Supporting Information Available

Materials and instrumentations; characterizations of HBPEC nanoparticles; cell-based assays including the MTT assay; cellular internalizations; ROS experiments; apoptosis and necrosis assays, and migration assays (PDF)
