# Supplementary material for: Core-Tunable Dendritic Polymer: A Folate-Guided Theranostic Nanoplatform for Drug Delivery Applications
Source: ACS Omega. 2024 Jul 6;9(28):30544–58. doi: 10.1021/acsomega.4c02258 (PMC11256300; doi:10.1021/acsomega.4c02258)
Supplement: Supplementary file 1 — ao4c02258_si_001.pdf [file ao4c02258_si_001.pdf]

“Supporting Information”

Core-Tunable Dendritic Polymer: Folate-Guided Theranostic Nanoplatform for Drug Delivery Applications

Neelima Koti,[1] Trishna Timalsena,[1] Kajal Kajal,[2] Caleb Worsley,[2] Adam Worsley,[2] Paul Worsley,[2] Carissa Sutton,[1] Tuhina Banerjee[1] and Santimukul Santra[1,*]

[1]Department of Chemistry and Biochemistry, Missouri State University, 901 S. National Avenue, Springfield, MO 65897, USA

[2]Department of Chemistry, Pittsburg State University, 1701 S. Broadway Street, Pittsburg, KS 66762, USA

*Corresponding author: Santimukul Santra (S.S.) Email: ssantra@missouristate.edu

Thermal property of HBPEC polymer by Differential Scanning Calorimetry (DSC).

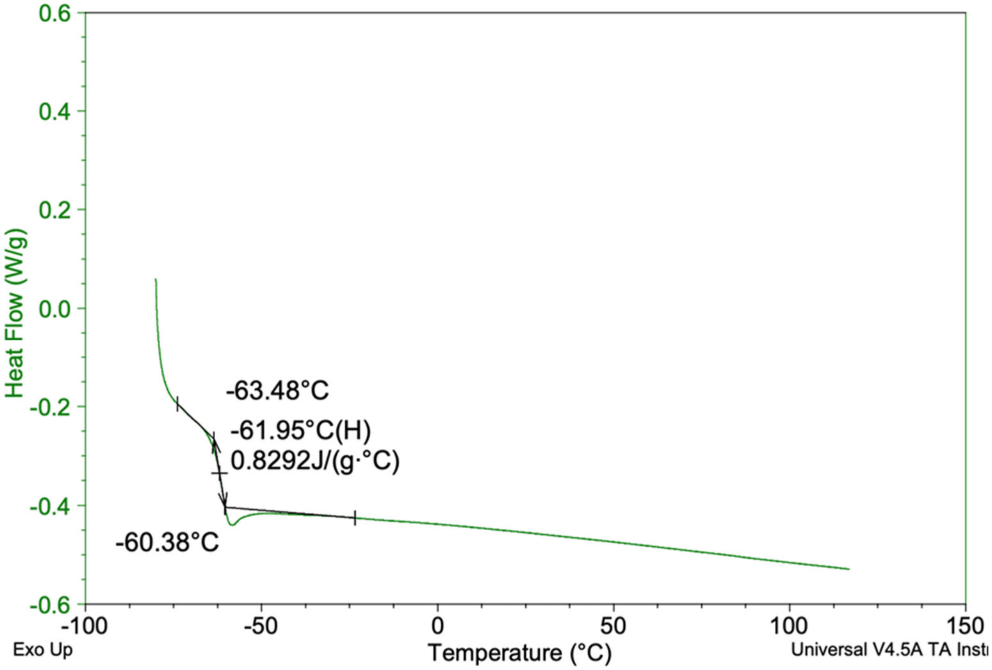

Figure S1: DSC scan of HBPEC polymer: Heat flow vs. temperature for the determination of thermal stability and crystallinity.

Characterizations for functional HBPEC nanoparticles (7 and 10).

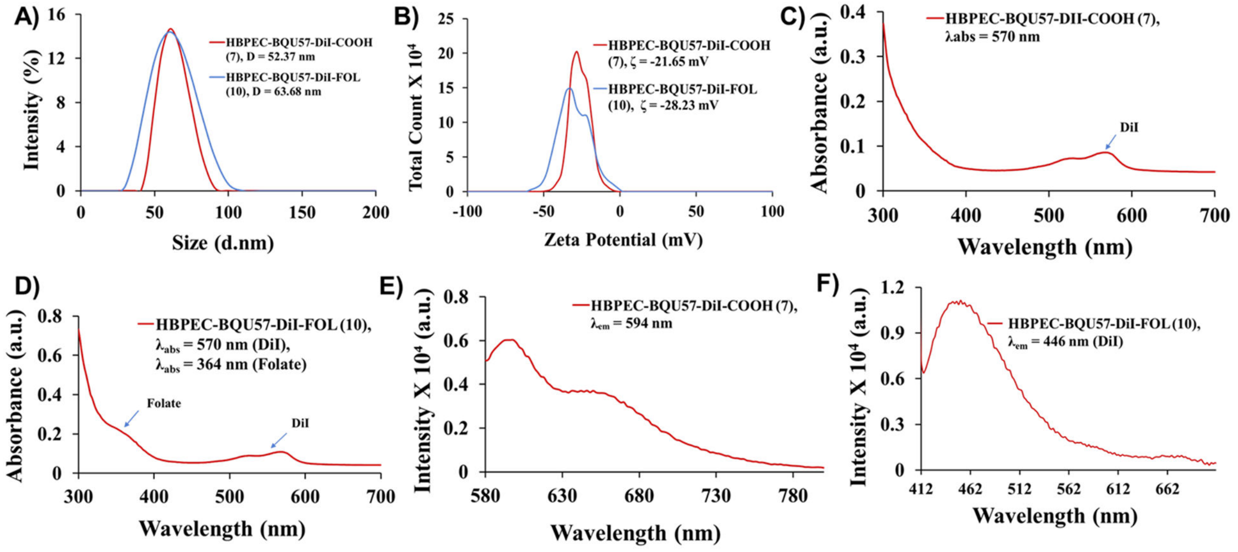

Figure S2: Spectroscopic analysis of DiI and BQU57 drug co-encapsulating (**7**) and folate conjugated (**10**) HBPEC nanoparticles. **A-B**) Dynamic light scattering (DLS) studies confirmed the formation of stable monodispersed HBPEC nanoparticles before and after folate conjugations. **C-F**) UV/Vis and fluorescence spectroscopic analyses further confirmed for the successful encapsulations.

***In vitro* cell-based assays for the assessment of HBPEC drug delivery systems.**

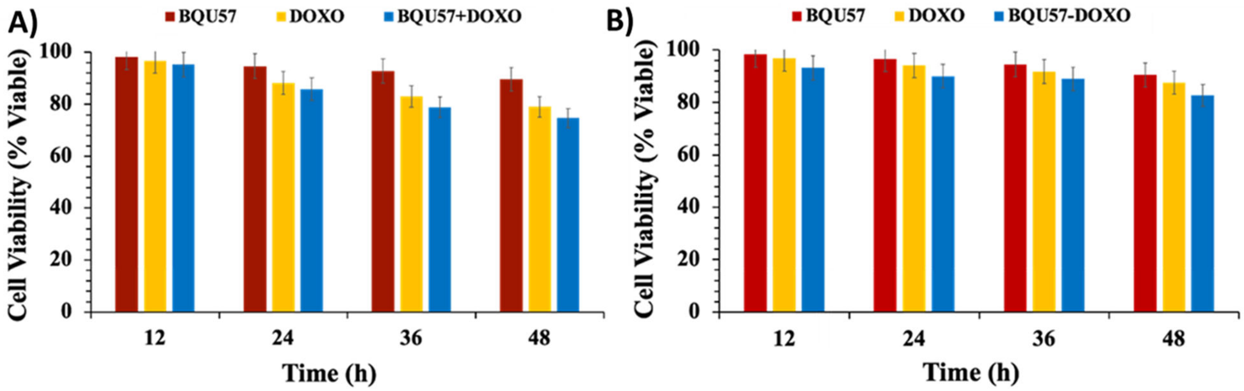

Figure S3: Cell viability experiments using folate negative H9c2 cells. **A)** Experiments were performed with HBPEC-folate nanoparticles (**10-12**) and **B)** non-folate HBPEC nanoparticles (**7-9**). Nominal cytotoxicity (15-25%) was observed in both the cases due to nominal expression of folate receptors on H9c2 cells and cellular internalizations. Experiments were performed in triplicate and calculated against standard error.

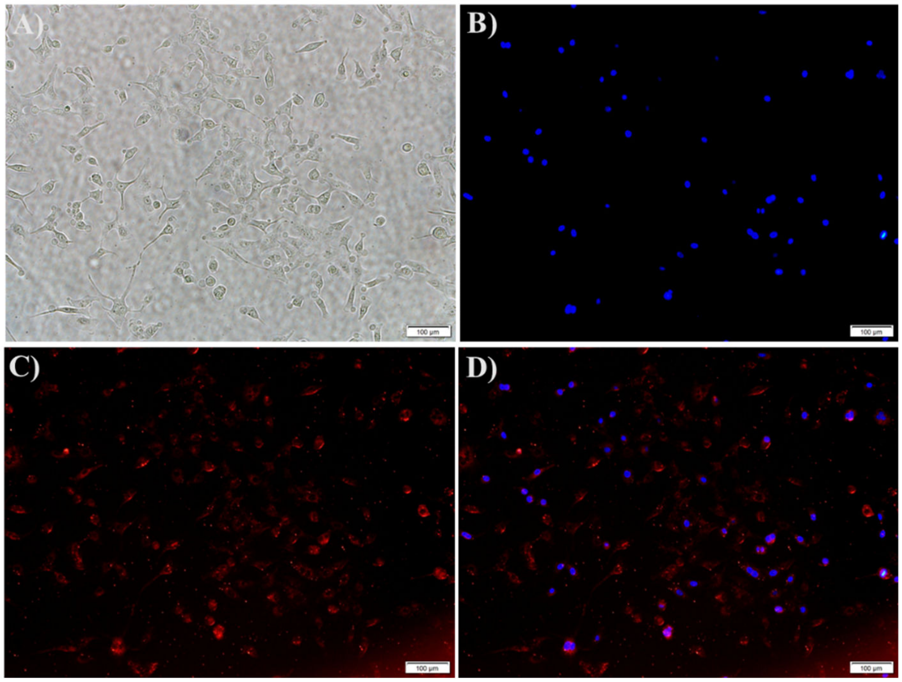

Figure S4: Fluorescence microscopic images of cellular internalization of HBPEC-BQU57-DII-FOL (**10**) nanoparticles in A549 cells. **A**) Brightfield image, **B**) nuclei stained with DAPI dye (blue filter), **C**) DiI dye fluorescence using red filter, and **D**) the merged image showing dye and nanoparticles' intracellular distribution, scale bar 100 μm.

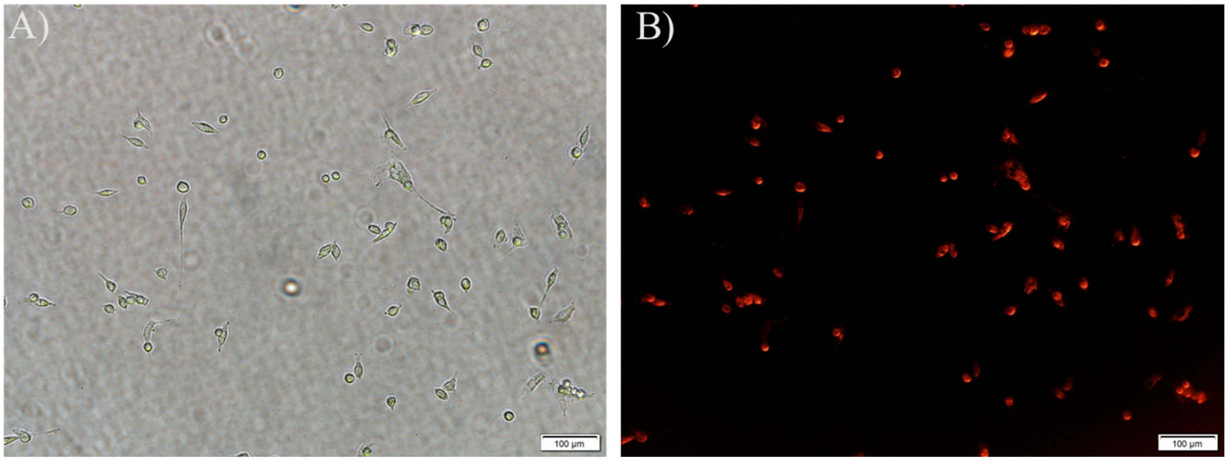

Figure S5: Determination of ROS in A549 cell line (scale bar 500 μm). **A**) Brightfield image of the generation of cytoplasmic ROS using HBPEC-BQU57-DiI-FOL (**10**), **B**) which are labeled with DHE dye (red filter, scale bar 100 μm).

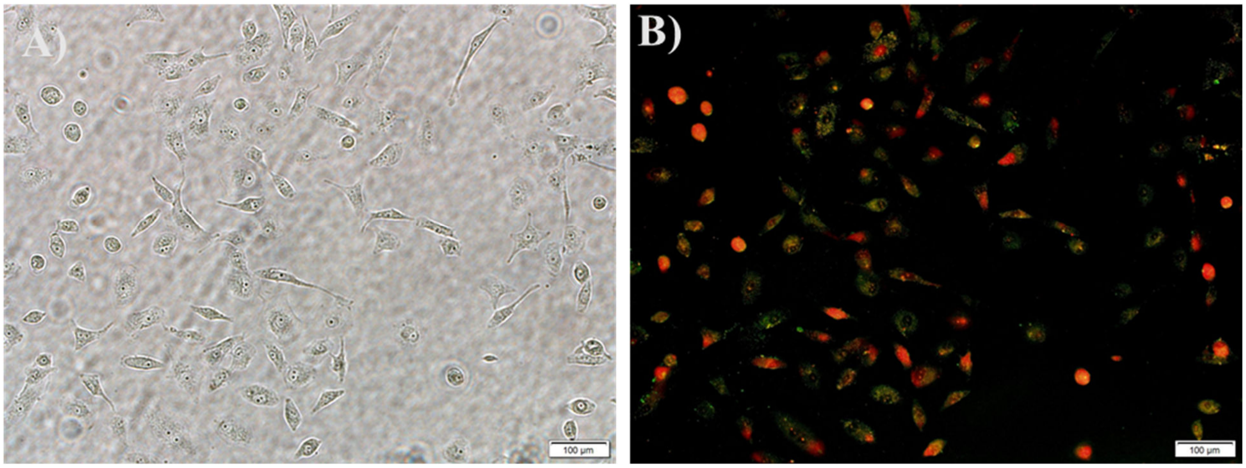

Figure S6: Determination of apoptosis and necrosis events in A549 cells after treatment with **A-B**) HBPEC-BQU57-DiI-FOL (**10**) for 24 h (scale bar 100 µm). A549 cells were then stained with Annexin V-FITC and Ethidium homodimer III dyes.

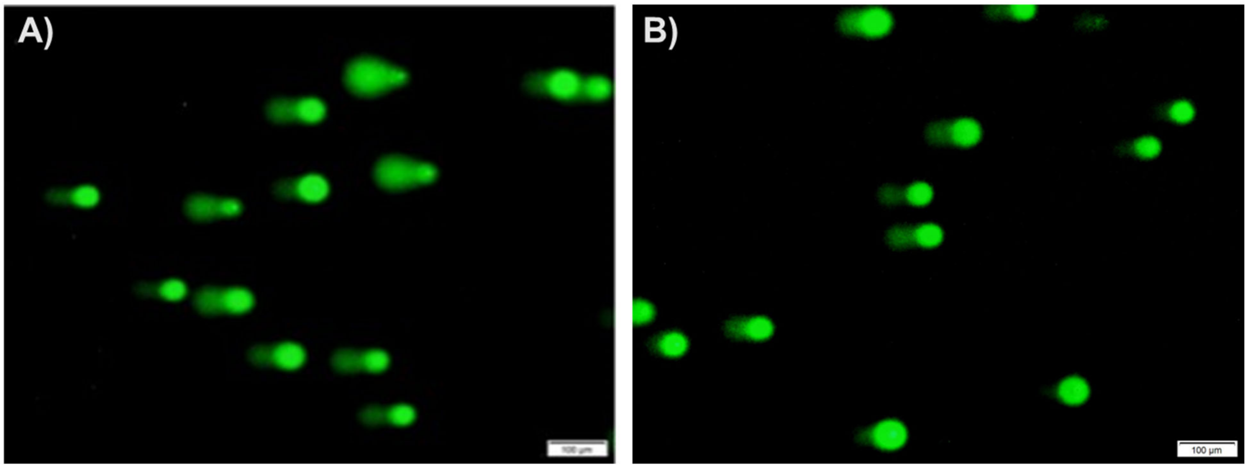

Figure S7: Comet assays were performed on the A549 cells using functional HBPEC nanoparticles. Representative microscopic images of the comet assay (**A**) in presence of HBPEC-DOXO-FOL nanoparticles (**11**) showing the formation of comet tails. (**B**) Experiment in presence of HBPEC-BQU57-DiI-FOL nanoparticles (**10**) showed no substantial formation of comet tails.
